# Cycloheximide congeners produced by *Streptomyces* sp. SC0581 and photoinduced interconversion between (*E*)*-* and (*Z*)-2,3-dehydroanhydrocycloheximides

**DOI:** 10.3762/bjoc.13.103

**Published:** 2017-05-30

**Authors:** Li Yang, Ping Wu, Jinghua Xue, Huitong Tan, Zheng Zhang, Xiaoyi Wei

**Affiliations:** 1 Key Laboratory of Plant Resources Conservation and Sustainable Utilization, Guangdong Provincial Key Laboratory of Digital Botanical Garden, South China Botanical Garden, Chinese Academy of Sciences, Xingke Road 723, Tianhe District, Guangzhou 510650, China; 2 University of Chinese Academy of Sciences, Yuquanlu 19A, Beijing 100049, China

**Keywords:** antifungal activity, cycloheximide derivatives, *E*/*Z* photoisomerization, *Streptomyces* sp, theoretical conformational analysis

## Abstract

Three new cycloheximide congeners, 2,3-dehydro-α-*epi*-isocycloheximide (**1**), (*E*)- and (*Z*)-2,3-dehydroanhydrocycloheximides (**2** and **3**), together with three known compounds, anhydroisoheximide (**4**), cycloheximide (**5**), and isocycloheximide (**6**), were obtained from the cultures of *Streptomyces* sp. SC0581. Their structures were elucidated by extensive spectroscopic analysis in combination with theoretical conformational analysis and ECD computations. The photoinduced interconversion between **2** and **3** was observed and verified and the possible reaction path and mechanism were proposed by theoretical computations. The antifungal and cytotoxic activities of **1**–**6** were evaluated and suggested that 2,3-dehydrogenation results in the loss of the activities and supported that the OH-α is important to the activities of cycloheximide congeners.

## Introduction

**Figure 1:**
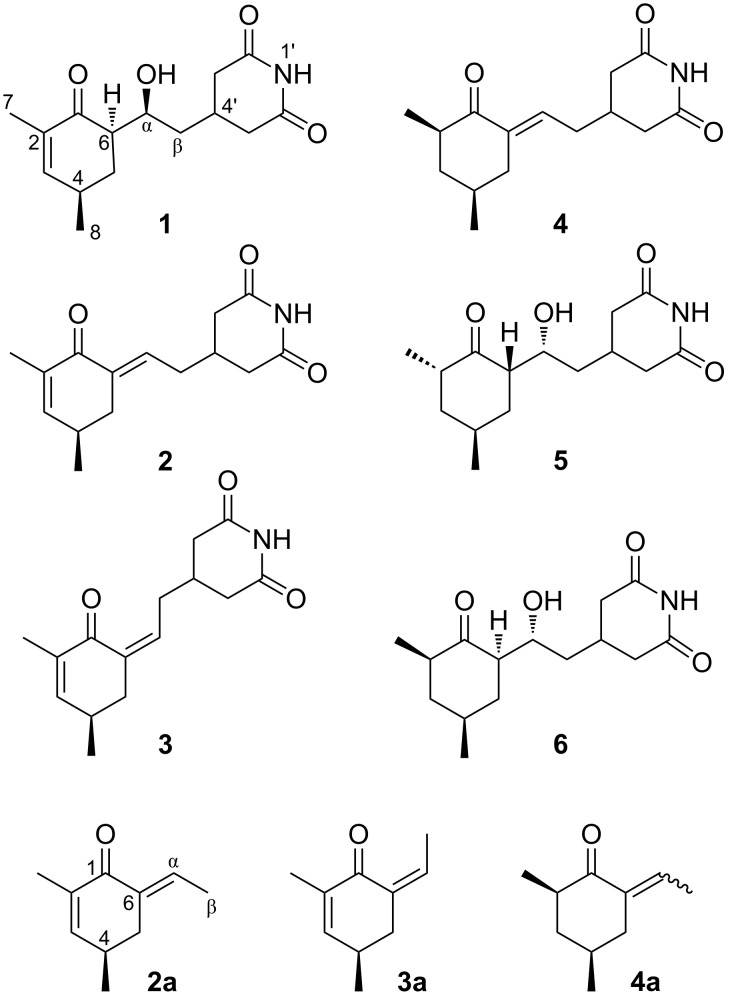
Structures of **1**–**6** and **2a**–**4a**.

**Figure 2:**
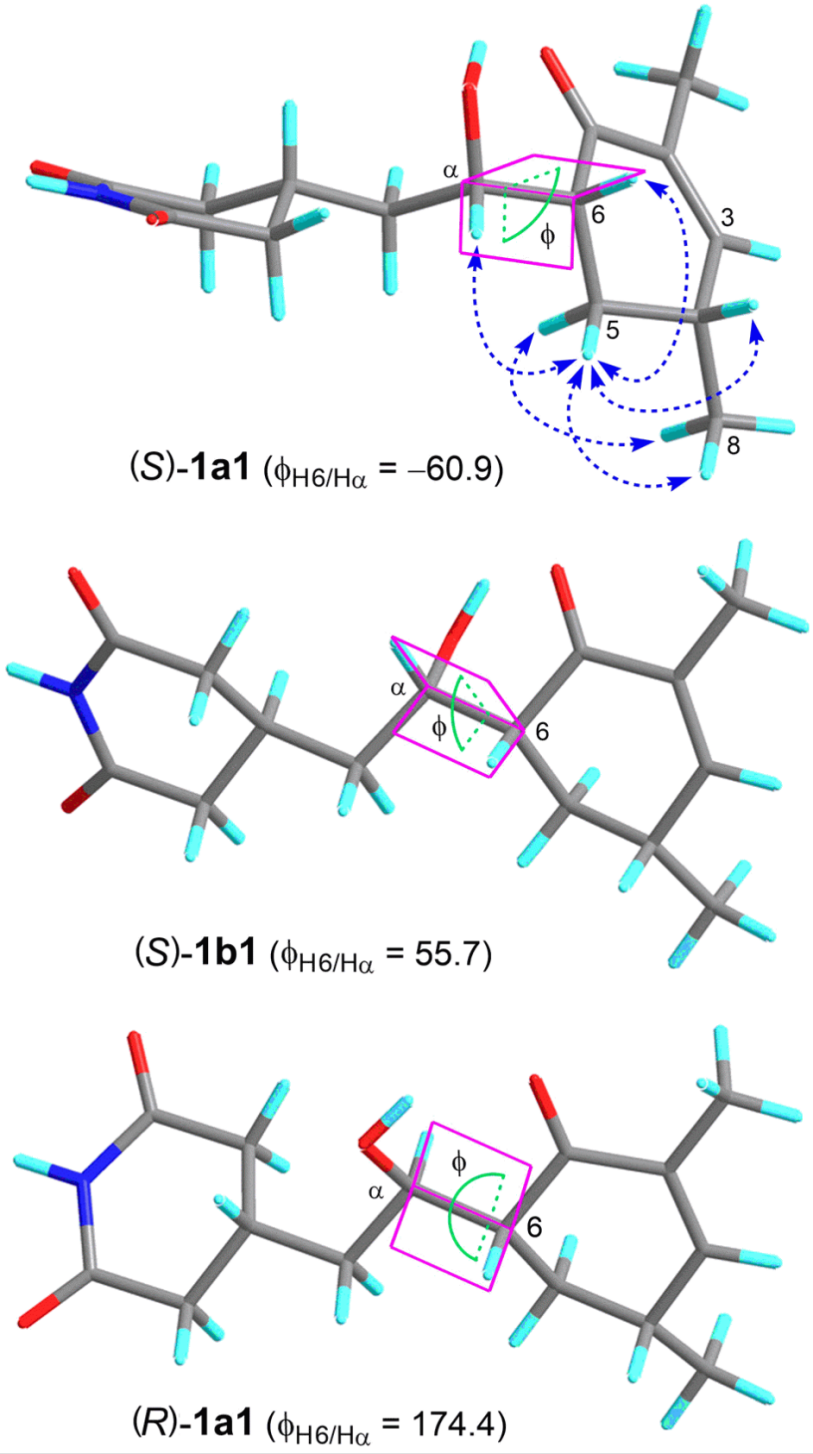
Representatives of the theoretical dominant conformers of (4*R*,6*R*,α*S*)-**1** ((*S*)-**1a1** and (*S*)-**1b1**) and (4*R*,6*R*,α*R*)-**1** ((*R*)-**1a1**) in equilibrium populations (in MeOH) and key NOESY correlations (dashed arrows) of **1**.

The glutarimide-containing antibiotics represent a fascinating class of natural products that exhibit a multitude of biological activities. The most famous representative of this family, cycloheximide (**5**), has been used for decades as an inhibitor of eukaryotic translation elongation [1–3]. Other members of this family show potent cell migration inhibition and antiviral activity [4–6], which continues to capture the attention from researchers in synthetic and biosynthetic chemistry, medicinal chemistry, and pharmacology. However, cycloheximide (**5**) has held back the clinical and agricultural applications due to its reproductive toxicity [7]. Identifying new analogues that offer similar activity without the toxic side-effects could provide a viable lead of therapeutic drugs or agricultural pesticides. During the course of our searching for bioactive microbial metabolites [8–9], a culture extract of *Streptomyces* sp. SC0581 was found to show antifungal activity against the phytopathogen *Phytophthora infestans*. Bioassay-guided fractionation of the extract led to the isolation of three new cycloheximide congeners (Figure 1), 2,3-dehydro-α-*epi*-isocycloheximide (**1**), (*E*)- and (*Z*)-2,3-dehydroanhydrocycloheximides (**2** and **3**), and one known but new naturally occurring cycloheximide congener, anhydroisoheximide (**4**) [6], together with cycloheximide (**5**) and isocycloheximide (**6**) [10]. Their structures were elucidated by spectroscopic analysis, theoretical conformational analysis, and ECD/TDDFT calculations. The photoinduced interconversion between **2** and **3** was observed and verified, and the possible reaction path and mechanism were proposed by theoretical computations. All the isolated compounds were evaluated for antifungal activity and cancer cell toxicity. Herein are reported the isolation, structural elucidation, and biological activities of these compounds and the interconversion between **2** and **3**.

## Results and Discussion

### Structure elucidation

The molecular formula of compound **1** was established as C_15_H_21_NO_4_ based on the HRESI(+)-MS ion at *m*/*z* 302.1357 [M + Na]^+^ (calcd 302.1363). Its ^1^H and ^13^C NMR spectral data (Table 1), in combination with the HSQC spectrum, indicated the presence of a conjugated keto carbonyl (δ_C_ 202.6), two amide carbonyls (δ_C_ 175.5, 175.7), a trisubstituted olefin (δ_H_ 6.65; δ_C_ 134.2, 151.9), two methyls (δ_H_ 1.72, 1.16; δ_C_ 14.5, 20.3), four aliphatic methines with one being oxygenated (δ_H_ 4.26; δ_C_ 67.3), and four methylenes. Analysis of the ^1^H,^1^H-COSY and HMBC spectra, in particular the HMBC correlations from the olefinic methyl protons (δ_H_ 1.72, H_3_-7) to C1 (δ_C_ 202.6), C2 (δ_C_ 134.2), and C3 (δ_C_ 151.9), from the aliphatic methyl (δ_H_ 1.16, H_3_-8) to C3, C4 (δ_C_ 31.8), and C5 (δ_C_ 31.7), and from the oxymethine (δ_H_ 4.26, H-α) to C1 and C6 (δ_C_ 52.2), readily constructed a planar structure of 2,3-dehydrocycloheximide.

**Table 1:** ^1^H (500 MHz) and ^13^C (125 MHz) NMR data of **1**–**3** in CD_3_OD.

	**1**			**2**			**3**	
					
position	δ_C_, type	δ_H_ (*J* in Hz)		δ_C_, type	δ_H_ (*J* in Hz)		δ_C_, type	δ_H_ (*J* in Hz)

1	202.6, C			190.5, C			192.8, C	
2	134.2, C			136.2, C			137.5, C	
3	151.9, CH	6.65 dq (3.7, 1.5)		153.4, CH	6.79 dq (3.6, 1.3)		152.3, CH	6.71 dq (3.2, 1.3)
4	31.8, CH	2.59 m		32.3, CH	2.59 m		32.2, CH	2.27 m
5	31.7, CH_2_	eq 2.16 dtd (12.7, 4.3, 1.8) ax 1.39 ddd (14.4, 12.7, 11.3)		34.6, CH_2_	β 2.86 dd (14.3, 5.1) α 2.30 m		43.3, CH_2_	β 2.74 dd (13.4,5.1) α 2.34 m
6	52.2, CH	2.54 dt (14.4, 4.3)		136.1, C			136.2, C	
7	14.5, CH_3_	1.72 dd (2.4, 1.4)		16.4, CH_3_	1.80 t (1.6)		16.1, CH_3_	1.76 t (1.6)
8	20.3, CH_3_	1.16 d (7.2)		21.0, CH_3_	1.14 d (7.9)		20.5, CH_3_	1.13 d (7.2)
α	67.3, CH	4.26 ddd (10.4, 4.3, 2.1)		134.6, CH	6.59 tt (7.6, 1.7)		137.1, CH	5.86 tt (7.7, 1.4)
β	37.2, CH_2_	1.58 ddd (14.0, 10.4, 3.6) 1.33 ddd (14.0, 8.2, 2.1)		33.4, CH_2_	2.31–2.42 m		34.5, CH_2_	2.56 m
2'	175.7, C			175.2, C			175.4, C	
3'	38.0, CH_2_	2.69 m 2.39 m		38.1, CH_2_	2.67 m 2.31–2.42 m		38.2, CH_2_	2.60–2.66 m 2.31–2.42 m
4'	27.4, CH	2.36 m		31.6, CH	2.31–2.42 m		33.8, CH	2.60–2.66 m
5'	36.5, CH_2_	2.79 m 2.37 m		38.2, CH_2_	2.60–2.66 m 2.31–2.42 m		38.3, CH_2_	2.60–2.66 m 2.31–2.42 m
6'	175.5, C			175.2, C			175.4, C	

**Figure 3:**
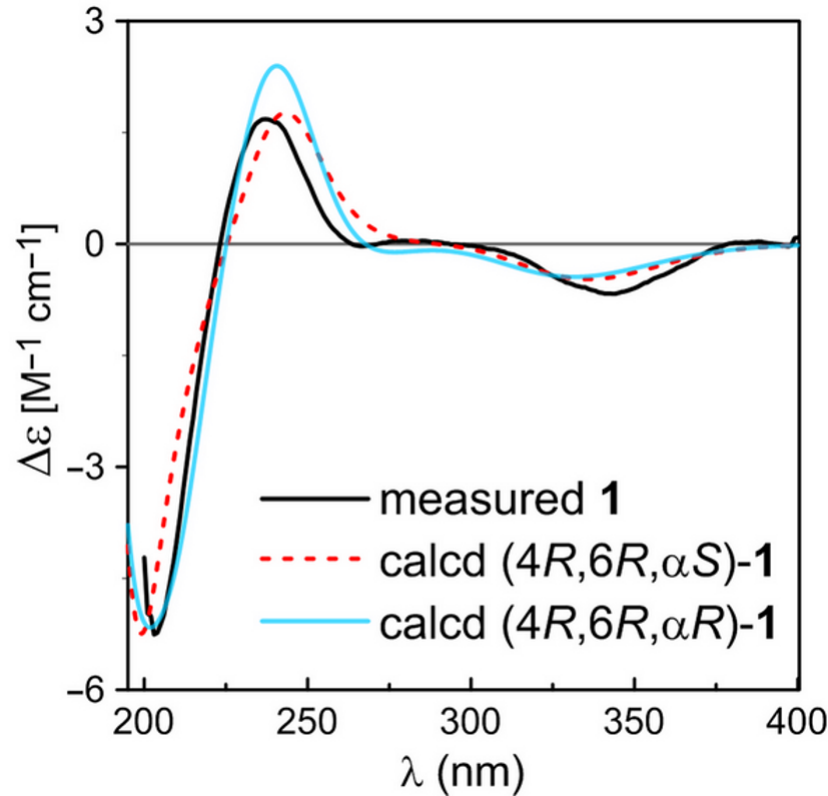
Comparison of the experimental ECD spectrum of **1** with the M11/TZVP calculated spectra of (4*R*,6*R*,α*S*)-**1** and (4*R*,6*R*,α*R*)-**1** in MeOH (σ = 0.38 eV for both; shift = +15 and +10 nm, scaling factor = 0.50 and 1.0, respectively).

The relative configuration of the cyclohexenone ring in **1** was assigned by analysis of the ^1^H NMR proton coupling constants (Table 1) and NOESY correlations. The large proton coupling constants, *J*_H6/H5ax_ = 14.4 Hz and *J*_H5ax/H4_ = 11.3 Hz, suggested that H6 and H4 were in pseudo-axial positions and on the same face of the cyclohexenone ring. This was further corroborated by the presence of strong NOE correlations of H_3_-8 with both H5ax and H5eq and of H5eq with both H6 and H4 in the NOESY spectrum (Figure 2). It was also in agreement with no NOE correlation being detected between H5ax and H6 or H4. However, straightforward analysis of the ^1^H NMR and NOESY spectra was unable to assign the relative configuration of Cα due to its location on the flexible side chain. In order to clarify the relative configuration of this chiral carbon, theoretical conformational analysis was carried out on two possible stereoisomers, (4*R*,6*R*,α*S*)-**1** and (4*R*,6*R*,α*R*)-**1**. MMFF conformational search and subsequent geometry optimization using the DFT-D3 method at the B3LYP-D3/6-31G(d) level followed by a higher level of energy calculations at the B3LYP-D3/def2-TZVP level afforded 14 and 11 distinctive low-energy conformers (Δ*G* < 2.5 kcal/mol) for (4*R*,6*R*,α*S*)-**1** and (4*R*,6*R*,α*R*)-**1**, respectively (Table S1, Supporting Information File 1). Among the low-energy conformers of (4*R*,6*R*,α*S*)-**1**, those having a *gauche* relationship between H-α/H-6 were found to be greatly dominant (accounting for about 96%) in the equilibrium mixture in MeOH, which could be categorized into two groups, (*S*)-**1a** and (*S*)-**1b**, based on the torsion angle of H6–C6–Cα–Hα (Φ_H6/Hα_) being around either −62° or +55° and each group constituted about 48% equilibrium populations. As can be seen in Figure 2, conformers in both (*S*)-**1a** and (*S*)-**1b** groups, represented by the second lowest energy minimum (*S*)-**1a1** (Δ*G* = 0.02 kcal/mol) and the global energy minimum (*S*)-**1b1**, respectively, matched up well with the proton coupling constant between H6/Hα (*J* = 4.3 Hz) and the aforementioned NOESY correlations. Furthermore, conformers in group (*S*)-**1a** were also consistent with the diagnostic NOE interaction of Hα/H5eq observed in the NOESY spectrum. In turn, (4*R*,6*R*,α*R*)-**1** afforded the dominant conformers (accounting for about 82% equilibrium populations), as represented by the global energy minimum (*R*)-**1a1** (Figure 2), having an *anti* relationship (Φ_H6/Hα_ ≈ 174°) between Hα/H6, which were inconsistent with the small *J*_H6/Hα_ value (4.3 Hz) and the absence of an NOE correlation between Hα/H5ax in the experimental spectra. Accordingly**,** the relative configuration of **1** was assigned to be 4R*,6R*,αS*. In order to determine the absolute configuration, the low-energy conformers of both stereoisomers were subjected to TDDFT calculations of the electronic circular dichroism (ECD) spectra. As shown in Figure 3, the calculated ECD spectra of (4*R*,6*R*,α*S*)-**1** and (4*R*,6*R*,α*R*)-**1** were similar to one another and both in good agreement with the measured spectrum of **1**, which indicated a *R* configuration of both C4 and C6 but gave no evidence for any of Cα. For the latter, the *S* configuration was assigned according to the relative configuration as deduced from above conformational analysis. Therefore, compound **1** was defined as 2,3-dehydro-α-*epi*-isocycloheximide.

**Figure 4:**
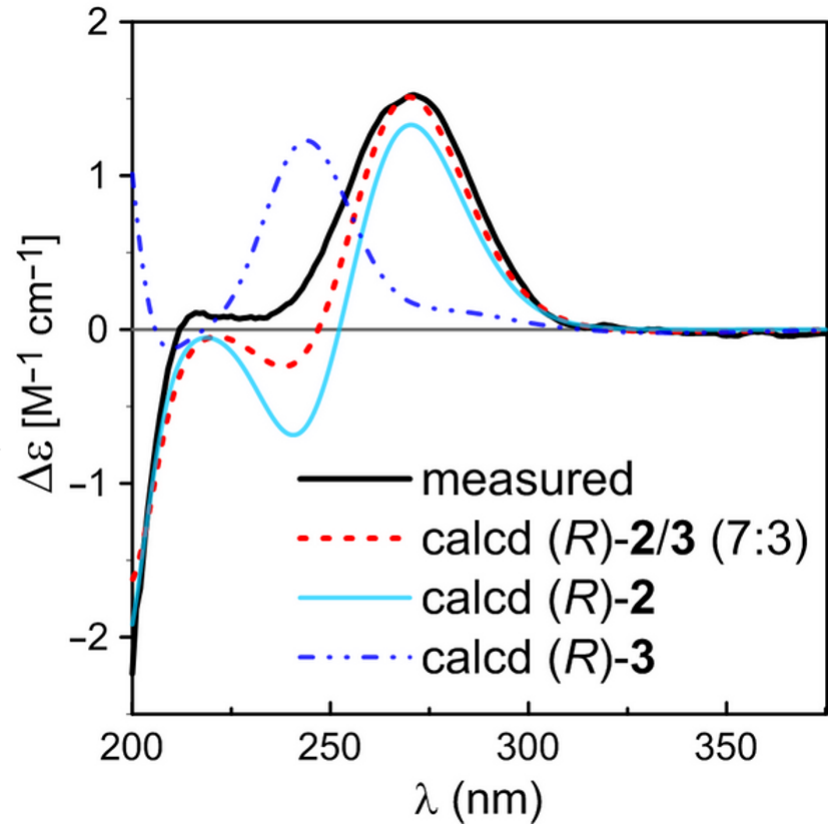
Comparison of the experimental ECD spectrum with the BH&HLYP/TZVP calculated spectra of the mixture of (*R*)-**2/3** (7:3) and individual (*R*)-**2** and (*R*)-**3** in MeOH (σ = 0.38 eV, shift = +18 nm, scaling factor = 0.44, 0.14, and 0.13, respectively).

Compounds **2** and **3** were obtained as a 7:3 equilibrium mixture. They could be separated by reverse phase HPLC using aqueous MeOH, but readily inter-converted and reached equilibrium in a couple of days at room temperature (in MeOH). The mixture gave a [M + Na]^+^ ion peak at *m*/*z* 284.1255 in the HRESI(+)-MS, appropriate for a molecular formula of C_15_H_19_NO_3_, having one H_2_O unit less than that of **1**. The 1D NMR spectra of the mixture contained two sets of signals in the ratio of 7:3 (according to the ^1^H NMR proton integral values), corresponding to two isomeric molecules **2** and **3** (Table 1), respectively. Each set of proton and carbon signals resembled those of compound **1** except for replacement of the resonances for the hydroxymethine at Cα and the aliphatic methine at C6 in **1** by the signals for an additional trisubstituted olefin [δ_H_ 6.59; δ_C_134.6 (CH), 136.1 (C) in **2** and δ_H_ 5.86; δ_C_137.1 (CH), 136.2 (C) in **3**], suggesting a gross structure of an anhydrous derivative of **1**. This was supported by the HMBC correlations of the olefinic proton Hα with C1 (δ_C_ 190.5 in **2**, 192.8 in **3**), C4' (δ_C_ 31.6 in **2**, 33.8 in **3**), and C5 (**2**, δ_C_ 34.6 in **2**, 43.3 in **3**). The structural difference between **2** and **3** was identified in the NOESY spectrum. The presence of a strong NOE correlation between the minor Hα (δ_H_ 5.86) and H5β (δ_H_ 2.74) was observed while the same correlation between the major counterparts [δ_H_ 6.59 (Hα), 2.86 (H5β)] could not be found in the spectrum, revealing the geometrical configurations of the double bond between C6/Cα were *E* in **2** and *Z* in **3**. In order to assign their absolute configurations, (*R*)-**2** and (*R*)-**3** were separately subjected to ECD/TDDFT calculations. As can be seen in Figure 4, the calculated ECD curve of (*R*)-**2** agreed well with the experimental spectrum of the mixture, whereas, that of (*R*)-**3** was largely inconsistent with the measured spectrum. Nevertheless, the summed ECD spectrum of (*R*)-**2** and (*R*)-**3** (7:3) showed the best fit with the experimental spectrum, indicating the *R* configuration of C4 in both compounds. Therefore, the structures of **2** and **3** were elucidated as (*E*)- and (*Z*)-2,3-dehydroanhydrocycloheximides, respectively. It is noted that a compound of unspecified absolute configuration was recently reported to possess the same planar structure as **3** [11].

### Photoinduced interconversion between **2** and **3**

**Figure 5:**
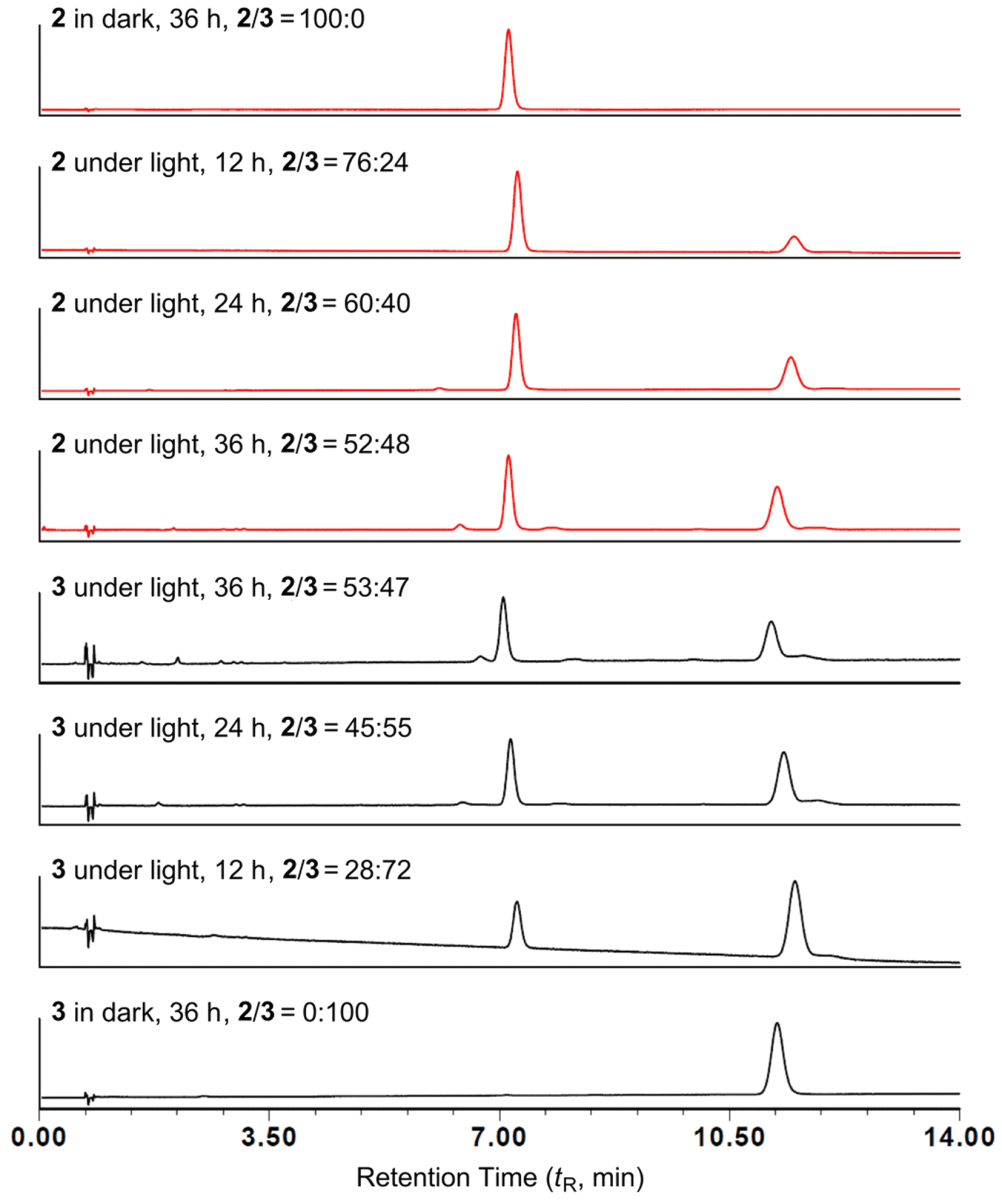
UPLC analysis of photoreaction products of **2** (around *t*_R_ = 7.5 min) and **3** (around *t*_R_ = 11.5 min).

When elucidating the structures of **2** and **3**, we understood that the interconversion between **2** and **3** is likely a photochemical process as it is known that most *Z*/*E* isomerizations of carbon double bonds in conjugated olefins and α,β-enones are facilitated by this process [12–14]. However, as all experiments in the present study were carried out either in the dark (fermentation) or under indoor light (extraction and fractionation), with neither direct sunlight shining into nor any artificial high-energy light being applied in the laboratory, we doubted if the fast conversion was induced by our laboratory indoor light. Thus, compounds **2** and **3** in the mixture were separated by preparative HPLC and their MeOH solutions were exposed to the natural indoor light at room temperature and subjected to UPLC analysis every 12 h. As a result, the interconversion between **2** and **3** was found to occur before 12 h and reach equilibrium (estimated ratios between **2**/**3** were 52:48 in **2** and 53:47 in **3** according to peak area intensities) at 36 h (Figure 5), whereas no changes were detected for the solutions kept in the dark (Figure 5), confirming the interconversion is a photoinduced geometrical isomerization.

**Figure 6:**
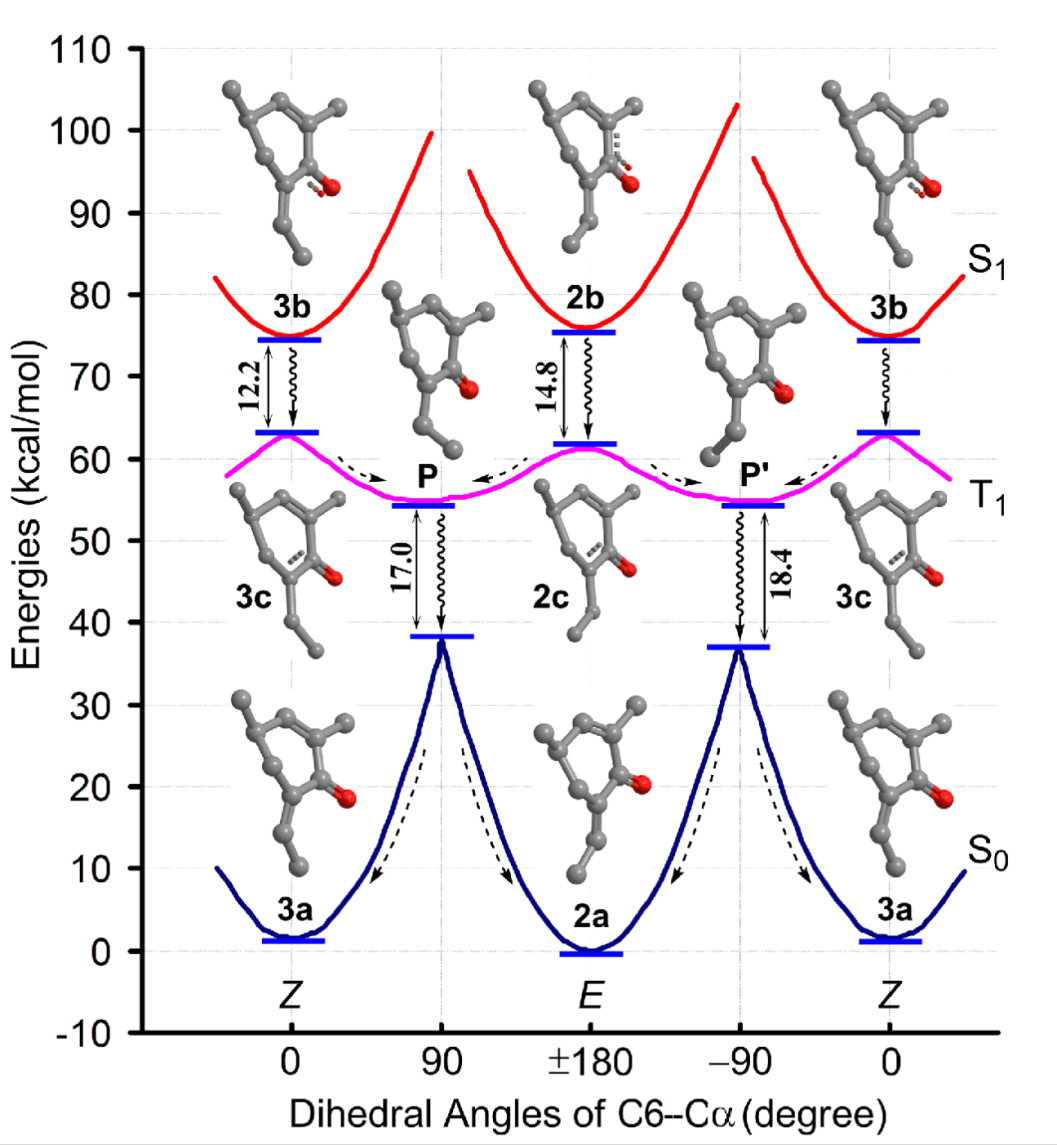
Potential energy surfaces of **2a**/**3a** in the S_0_, S_1_, and T_1_ states, geometries of key points in the surfaces, and proposed photoreaction path (dashed arrow: vibrational relaxation; wavy arrow: ISC; double headed arrow: energy gap between the two points).

Compounds **2** and **3** possess a chiral 6-ethylidene-2-cyclohexenone chromophore. To the best of our knowledge, *Z*/*E* photoisomerizations of compounds with this kind of chromophore had been rarely reported. For better understanding the insights into this reaction, we conducted theoretical investigation using the truncated structures **2a** and **3a** (Figure 1). At first, the lowest-energy geometries of **2a** and **3a** in MeOH in the ground (S_0_), first singlet excited (S_1_), and first triplet excited (T_1_) states were optimized at the B3LYP/def2-SVP level. Starting from these energy minima, other equilibrium points rotating around the C6–Cα double bond in S_0_, S_1_, and T_1_ states were located by relaxed potential energy surface scans, except for geometries around torsion angles (C1–C6–Cα–Cβ, Φ) of 90° and −90° in the S_1_ surface, for which optimizations failed to converge. Due to the presence of the stereogenic center C4 in **2a** and **3a**, the twisted structures around Φ 90º and −90° are diastereomeric in chirality [15]. Thus, two twisted minima, P and P′ (Φ = 77º and −84°, respectively) in the T_1_ surface and two energy maxima (Φ = 90° and −90°, respectively) in S_0_ surface were located. The resultant potential energy surfaces of the three states and the geometries of key stationary points are shown in Figure 6. The key bond lengths and torsion angles Φ of these geometries are presented in Table 2. Compound **4** has a structure similar to **2** except for replacement of the C2–C3 double bond by a single bond. Its geometrical isomerization was not observed in the present study, but (*E*)-2-ethylidenecyclohexanone, which has a similar chromophore to that of **4**, was reported to be able to give the *Z*-isomer upon photoirridiation [16]. For comparison purpose, the truncated structure (**4a**, Figure 1) of this compound was also calculated using the same methods. The lowest-energy geometries of (*E*)- and (*Z*)-**4a** in the S_0_, S_1_ ((*E*)-**4a**-S_1_, (*Z*)-**4a**-S_1_), and T_1_ (P-**4a**, P′-**4a**) surfaces and the two energy maxima ((*E*)-**4a**-T_1_, *(Z*)-**4a**-T_1_) in the T_1_ surface were optimized and their key geometrical parameters are also listed in Table 2. Analysis of geometrical parameters of key points in the excited and ground states (Table 2) showed that the S_1_ and T_1_ minima of **2a**/**3a** correspond to stable ^1^(n–π*) and ^3^(π–π*) states of α,β-enones [14], respectively, and the T_1_ planar maxima, with increased the C1–O1 and C6–Cα bond lengths and a decreased C1–C6 bond length relative to those of T_1_ minima, show similarity to a T_2_ π–π* species [14]. Comparison of parameters of geometries in excited states between **2a**/**3a** and **4a** revealed that the electron delocalization are extended to C2 and C3 in the S_1_ minima **2b** and **3b** and due to this, their C1–C6 bond length is increased by about 0.04 Å while that of C6–Cα is decreased by about 0.03 Å with respect to those in (*E*)-**4a**-S_1_ and (*Z*)-**4a**-S_1_ (Table 2), implying that the presence of C2–C3 double bond in **2a**/**3a** strengthens C6–Cα double bond and increases the rotational barrier of C6–Cα bond in the S_1_ (n–π*) state. In contrast, no significant differences between **2a**/**3a** and **4a** can be found in the T_1_ (π–π*) states, including the rotational barriers of C6–Cα bond (Table 2).

**Table 2:** Relative energies and selected geometrical parameters of key stationary points in S_0_, S_1_, and T_1_ potential energy surfaces of **2a**/**3a** and **4a**.

	Δ*E*	bond lengths (Å)						Φ
		
geometry	(kcal/mol)	C1–O1	C1–C2	C2–C3	C1–C6	C6–Cα	Cα–Cβ	(degrees)

**2a**	0.0	1.228	1.492	1.351	1.500	1.350	1.494	−179.94
**3a**	1.57	1.229	1.494	1.352	1.496	1.352	1.497	0.72
**2b**	75.73	1.308	1.445	1.369	1.449	1.372	1.495	176.99
**3b**	74.70	1.308	1.452	1.367	1.449	1.369	1.491	−0.54
P	54.85	1.250	1.491	1.347	1.452	1.466	1.498	76.78
P′	54.80	1.250	1.491	1.347	1.452	1.466	1.498	−83.71
**2c**	61.18	1.266	1.486	1.352	1.429	1.497	1.482	177.72
**3c**	62.88	1.263	1.492	1.351	1.431	1.503	1.483	−1.171

*(E*)-**4a**	0.0	1.222	1.532	1.536	1.505	1.351	1.494	−179.95
*(Z*)-**4a**	1.40	1.222	1.533	1.540	1.501	1.351	1.497	1.04
*(E*)-**4a**-S_1_	77.06	1.291	1.552	1.530	1.410	1.400	1.494	177.96
*(Z*)-**4a**-S_1_	76.10	1.292	1.557	1.530	1.412	1.396	1.490	1.41
P-**4a**	53.19	1.241	1.535	1.535	1.456	1.469	1.495	79.08
P′-**4a**	53.19	1.241	1.535	1.535	1.456	1.469	1.495	83.24
*(E*)-**4a**-T_1_	60.0	1.263	1.533	1.535	1.428	1.505	1.484	−175.70
*(Z*)-**4a**-T_1_	61.0	1.254	1.539	1.533	1.436	1.505	1.486	4.30

In respect to the photoreaction path, it is predicted that the *Z*/*E* isomerization of **2a**/**3a** is impossible to take place along the S_1_ surface due to the presence of large rotational barrier (>25 kcal/mol) in accessing the twisted intermediates (Figure 6). Instead, radiationless relaxations via an intersystem crossing (ISC) from the S_1_ minima **2b** and **3b** to the T_1_ maxima **2c** and **3c**, respectively, are energetically and symmetrically reasonable [14,17] as well as are preferred according to El-Sayed’s rules [18]. Therefore, the isomerization of **2a**/**3a**, after vertical excitation, is proposed to proceed in the route as shown in Figure 6, which involves radiationless decay from the S_1_ minima to the triplet manifold via an ISC and then from the T_1_ twisted minima to the ground state via a second ISC, leading to isomerization of the C6–Cα double bond. This route is generally similar to those proposed for isomerization of the C–C double bond in acyclic α,β-enones [14,17], but more complicated in details. Due to the presence of two T_1_ minima and two S_0_ maxima, there are two equivalent points for the second ISC, one around Φ = 90° the other around Φ = −90°, and two points being with slightly different energy gaps. Any of these two ISC points is accessible from either **2c** or **3c** and can decay to either **2a** or **3a** (Figure 6).

With the calculated energies, some observed results in experiments can also be explained. The slightly faster reaction of **3** and higher yield of **2** in equilibrium shown in Figure 5 are generally in accordance with the difference in energies between **2a** and **3a** (Table 2). They may also be related to the energy gaps between **2b**/**2c** and **3b**/**3c** (Figure 6), for which a more efficient ISC is expected for the smaller energy gap [14,17]. The weak reactivity of **4** in photoisomerization (not observed in the present study) can be attributable mainly to its higher vertical excitation energy (87.1 kcal/mol in **4a**) relative to those of **2** (81.6 kcal/mol in **2a**) and **3** (79.5 kcal/mol in **3a**).

### Antifungal activity and cytotoxicity

Compounds **1** and **4**–**6** and the mixture of **2** and **3** were evaluated for the in vitro antifungal activity against *Colletotrichum gloeosporioides*, *Phytophthora infestans*, and *Saccharomyces cerevisiae* using a previously described method [19]. The minimum inhibitory concentration (MIC) data were listed in Table 3. Compounds **5** and **6**, as previously reported [20–21], displayed activity against all tested fungal strains and **5** showed a superior activity to **6**, whereas compounds **1** and **4** and the mixture of **2** and **3** were found to be inactive (MIC > 100 μg/mL) against all tested strains, which supported the previous finding that the OH-α was important to the antifungal activity of cycloheximide derivatives (**4** vs **6**) [21–22], and suggested that 2,3-dehydrogenation might result in the loss of the activity (**1** vs **5**, **6**). The cytotoxicity of these isolates against human lung epithelial carcinoma (A549), human cervical epithelial adenocarcinoma (HeLa), and human breast carcinoma (MCF-7) was also evaluated, using MTT assay [23] with doxorubicin as a positive control. As shown in Table 3, the isolates showed a similar activity profile with that of the antifungal activity, e.g., only **5** and **6** were active and others were inactive (>50 μM) toward all the tested cell lines. These findings suggested that these cycloheximide derivatives possibly exert the antifungal and cytotoxic activities via a similar mode of action.

**Table 3:** Antifungal activity and cytotoxicity of **1**–**6**^a^.

	antifungal activity (MIC, μg/mL)				cytotoxicity (IC_50_, μM)^b^		
			
compound	*C. gloeosporioides*	*P. infestans*	*S. cerevisiae*		A549	Hela	MCF-7

**5**	25.0	25.0	3.1		15.8 ± 3.4	15.6 ± 3.6	18.1 ± 0.2
**6**	50.0	50.0	6.3		30.0 ± 3.8	21.9 ± 2.9	21.9 ± 2.9
doxorubicin					0.12 ± 0.02	0.31 ± 0.02	1.00 ± 0.20

^a^Compounds **1** and **4** and the mixture of **2** and **3** being inactive against all tested fungi (MIC > 100 μg/mL) and tumor cells (IC_50_ > 50 μM), are not listed. ^b^Values represent means ± SD based on three individual experiments.

## Conclusion

Three new (**1**–**3**) and three known (**4**–**6**) cycloheximide congeners were obtained from the cultures of *Streptomyces* sp. SC0581. The structure elucidation of the new compounds were achieved by spectroscopic analysis in combination with theoretical conformational analysis and ECD simulations, in which theoretical computations were shown to play a key role in solving challenges in assignments of relative and absolute configurations. Analysis of the antifungal and cancer cell toxic activity data of **1**–**6** suggested that change of the C2–C3 single bond to a double bond can lead to the loss of the activities and supported the OH-α is important to the activities of cycloheximide congeners. Furthermore, compounds **2** and **3**, with a chiral 6-ethylidene-2-cyclohexenone chromophore, were found to undergo *E*/*Z* photoisomerization under the indoor light. Theoretical investigation showed the presence of the C2–C3 double bond in **2** and **3** strengthens the C6–Cα double bond and increases the rotational barrier of the C6–Cα bond in the S_1_ (n–π*) states due to the extended electron delocalization, whereas it scarcely affects the T_1_ (π–π*) states, compared to those in **4**. It also revealed a photoisomerization route similar to that commonly found in acyclic α,β-enones [14,17], except for the presence of two equivalent points (around Φ 90° and −90°, respectively) for the ISC from T_1_ to S_0_ state, arising from the chiral nature of molecules.

## Experimental

### General experimental procedures

Optical rotations were recorded in MeOH on a Perkin-Elmer 343 spectropolarimeter. UV spectra and ECD spectra were obtained simultaneously on a Chirascan CD spectrometer (Applied Photophysics Ltd., England) using MeOH as solvent. ^1^H NMR, ^13^C NMR, and 2D NMR data were recorded on a Bruker Avance III 500 MHz spectrometer with TMS as internal standard. HRESIMS data were recorded on a Bruker maXis Q-TOF spectrometer. Preparative HPLC were carried out with a Shimadzu Shim-packed Pro-ODS column (20 mm × 25 cm) equipped with a Shimadzu LC-6AD pump and a Shimadzu RID-10A refractive index detector. UPLC analysis was performed on an Acquity H-Class UPLC system consisting of a quaternary solvent delivery system, an auto-sampler, and a DAD detector. For column chromatography, silica gel 60 (100–200 mesh, Qingdao Marine Chemical Ltd., Qingdao, People's Republic of China), YMC ODS (75 μm, YMC Co. Ltd., Kyoto, Japan) and Sephadex LH-20 (GE Healthcare, Uppsala, Sweden) were used. Analytical TLC were performed on HSGF254 silica gel plates (0.2 mm, Yantai Jiangyou silica gel Development Co. Ltd., Yantai, China); spots were visualized after spraying with 10% H_2_SO_4_ solution followed by heating.

### Biological material

*Streptomyces* sp. SC0581, isolated from a soil sample collected from Dinghu Mountain Biosphere Reserve, Guangdong, People's Republic of China, was identified according to morphological characteristics and sequence analysis of the ITS region (GenBank accession no. KX687558). A reference strain maintained at −80 °C was deposited in the culture collection of South China Botanical Garden, Chinese Academy of Sciences, Guangzhou, People's Republic of China.

### Fermentation, extraction and isolation

The strain was grown on the PDA medium at 28 °C for 10 days and 6 pieces of PDA culture plugs of the strain were inoculated into each of two 500 mL Erlenmeyer flasks containing 150 mL of seed medium (glucose 0.4%, malt extract 1.0%, yeast extract 0.4%, pH 5.5) and shaken on a rotatory (150 rpm) at 28 °C for 2 days. Then, the cultures were transferred into three 3 L flasks containing 1 L of seed medium and cultivated at the same culture conditions. Finally, 10 mL each of the culture broth was transferred into two hundred 500 mL flasks containing 60 mL of YMG medium and 60 g of wheat grains, and the fermentation was carried out stably in the dark at 28 °C for 40 days. The obtained solid fermentation cultures were extracted with 95% EtOH for three times and concentrated in vacuum. The resultant extract was successively partitioned between petroleum ether, EtOAc, and *n*-BuOH. The EtOAc and *n*-BuOH soluble fractions, showing the activity against *P. infestans*, were combined and subjected to silica gel column chromatography (CC) eluted with CHCl_3_/MeOH mixtures of increasing polarity (100:0 to 70:30) to give twenty fractions including two antifungal fractions, Fr. 1 (CHCl_3_/MeOH, 95:5) and Fr. 6 (CHCl_3_/MeOH, 90:10). Fr. 1 (6.0 g) was further separated by ODS CC using aqueous MeOH (10–90%) to obtain five subfractions (Frs. 1A–1E). Fr. 1B (40% MeOH) was purified by preparative HPLC using isocratic elution of aqueous CH_3_CN (20%) at a flow rate of 5 mL/min to afford **5** (9 mg, *t*_R_ = 80.4 min), **6** (5 mg, *t*_R_ = 60.0 min), and the mixture of **2** and **3** (4 mg, **2**, *t*_R_ = 105.5 min, **3**, *t*_R_ = 180.2 min). Fr. 1C (50% MeOH) was separated by preparative HPLC using 40% MeOH at 5 mL/min to yield **4** (15 mg, *t*_R_ =110.8 min). Fr. 6 (4.4 g) was separated by ODS CC using aqueous MeOH (10–90%) followed by preparative HPLC using 40% MeOH at 5 mL/min to give **1** (10 mg, *t*_R_ = 108.6 min).

**2,3-Dehydro-α-epiisocycloheximide (1):** Colorless oil; [α]_D_^25^ +1.8 (*c* 0.20, MeOH); UV (MeOH) λ_max_ (log ε) 201 (3.40), 237 (3.14); CD (MeOH) λ(∆ε) 203 (−5.06), 237 (+1.62), 344 (−0.85); ^1^H and ^13^C NMR data, see Table 1; HRESIMS (*m*/*z*): [M + Na]^+^ calcd for C_15_H_21_NNaO_4_, 302.1363; found: 302.1357.

**Mixture of (*E*)-2,3-dehydroanhydrocycloheximide (2) and (*Z*)-2,3-dehydroanhydrocycloheximide (3):** Colorless oil; [α]_D_^25^ +25.5 (*c* 0.25, MeOH); UV (MeOH) λ_max_ (log ε) 204 (4.04), 244 (3.58), 272 (3.49); CD (MeOH) λ(∆ε) 271 (+1.58); ^1^H and ^13^C NMR data, see Table 1; HRESIMS (*m/z*): [M + Na]^+^ calcd for C_15_H_19_NNaO_3_, 284.1257; found: 284.1255.

### Evaluation of in vitro antifungal activity

*Saccharomyces cerevisiae*, *Colletotrichum gloeosporioides*, and *Phytophthora infestans* were used as the test fungal strains. The antifungal activity was evaluated using the microplate Alamar Blue assay (MABA) [19]. For the filamentous fungi, *C. gloeosporioides* and *P. infestans*, the strains were grown on PDA cultures for 7 days. The spore suspensions were harvested by flooding the colony with PDB medium, rubbing the surface with a sterile scraper, and filtering with four layers of gauze. The spore suspensions were subjected to quantification with a hemocytometer and adjusted with PDB medium to 1 × 10^5^ CFU/mL for the microplate Alamar Blue assay. Test compounds were diluted with the DMSO to give two-fold gradient concentrations. 100 μL of spore suspension of each strain containing Alamar Blue (8%, v/v) and the compound solution (4%, v/v), was added into 96-well microtiter plate in triplicate. The final concentrations of tested compounds were 100, 50, 25, 12.5, 6.25 and 3.125 μg/mL. To negative control wells were added DMSO instead of the test compound, and blank control wells contained Alamar Blue but without spore suspension. The plate was incubated in the dark at 28 °C for 6–8 hours. When the color of negative control wells switched from blue to red, the final concentration of the well which was closest to the red one and remained blue was received as the minimal inhibitory concentration (MIC). With regard to the *S. cerevisiae,* the strain was incubated in PDB medium on a rotary shaker at 150 rpm in 28 °C for 12 hours. The suspensions of the strain were quantified with a hemocytometer and adjusted with PDB medium to 1 × 10^4^ CFU/mL. The next steps of microplate Alamar Blue assay for *S. cerevisiae* were the same as mentioned above for the two filamentous fungi.

### Evaluation of tumor cell toxicity

The evaluation was conducted as previously described [23].

### Photoinduced interconversion between **2** and **3**

The mixture of **2** and **3** (7:3) was separated by a SPOLAR C18 column (4.6 × 250 mm, 5 μm) using 35% MeOH at a flow rate of 1 mL/min, yielding compounds **2** (*t*_R_ = 19.3 min) and **3** (*t*_R_ = 28.5 min) in pure form. The whole separation process was performed in the dark. Then, two aliquots of MeOH solutions were prepared for each of **2** and **3**. One aliquot of solution was placed under the natural indoor light (illumination ranging between 155–315 lux) at room temperature and the other was kept in the dark. The two aliquots of solutions were subjected to UPLC analysis every 12 hours on an Acquity UPLC BEH C18 column (2.1 × 100 mm, 1.7 μm) using 35% MeOH as mobile phase at a flow rate of 0.3 mL/min. The column temperature was controlled at 40 °C and detection wavelength was set at 280 nm.

### Computational details

Molecular Merck force field (MMFF) calculations were done using the Spartan'14 program (Wavefunction Inc., Irvine, CA, USA). Density functional theory (DFT) and time-dependent density functional theory (TDDFT) calculations were performed with the Gaussian 09 program package [24]. For computations of ECD spectra, the conformers generated by a MMFF conformational search in an energy window of 10 kcal/mol were subjected to geometry optimization using the dispersion-corrected DFT-D3 method [25] at the B3LYP-D3/6-31G(d) level. Frequency calculations were carried out on those optimized conformers with relative energies (Δ*E*) less than 4.5 kcal/mol using the same level to verify that they were true minima and to estimate their relative thermal free energies (Δ*G*) at 298.15 K. The more accurate energies of these conformers in MeOH were obtained with the B3LYP-D3/def2-TZVP method. Solvent effects were taken into account by using polarizable continuum model (PCM). The TDDFT calculations were performed using the hybrid BHandHLYP [26], M11 [27], and/or PBE1PBE [28–29] functionals, and Ahlrichs’ basis set TZVP (triple zeta valence plus) polarization [30]. The number of excited states per each molecule was 24. The ECD spectra were generated by the program SpecDis [31] using a Gaussian band shape from dipole-length dipolar and rotational strengths. Equilibrium population of each conformer at 298.15 K was calculated from its relative free energies using Boltzmann statistics. The calculated spectra were generated from the low-energy conformers according to the Boltzmann weighting of each conformer in MeOH solution.

For theoretical investigations on the photoinduced interconversion between **2** and **3**, the truncated structures **2a** and **3a** (Figure 1) were used. Geometries of **2a** and **3a** in MeOH solution in S_0_, S_1_, and T_1_ states were optimized by DFT (for S_0_ and T_1_) or TDDFT (for S_1_ only) calculations at the B3LYP/def2-SVP level. For diradical triplets, the spin-unrestricted formalism was used. Solvent effects were treated using PCM. To build 3D conformers of **2a** and **3a**, the global energy minima of **2** and **3**, obtained in above ECD computations, were used and their glutarimide ring was replaced by a hydrogen atom. The built conformers were subjected to geometry optimizations to obtain the absolute energy minima of **2a** and **3a** in the S_0_, S_1_, and T_1_ states. Starting from these minima, other equilibrium points rotating around the C6–Cα double bond in surfaces of the three states were located by relaxed potential energy surface scans, except for those around Φ 90° and −90° in the S_1_ surface, for which optimizations failed to converge. Frequency calculations were also carried out on the significant points, including geometries **2a**–**2c**, **3a**–**3c**, P, and P′ and verified that they were either true minima or maxima (**2c** and **3c** only). Structures (*E*)- and (*Z*)-**4a** were calculated with the same method and their stable energy minima in the S_0_, S_1_, and T_1_ states and T_1_ maxima were optimized (Table 2).

## Supporting Information

File 1 HRESIMS, 1D and 2D NMR spectra of compounds **1**–**3**; energies, populations, and key torsion angles of low-energy conformers of compounds **1**–**3** by theoretical computations.
