# Supplementary material for: Cycloheximide congeners produced by Streptomyces sp. SC0581 and photoinduced interconversion between (E)- and (Z)-2,3-dehydroanhydrocycloheximides
Source: Beilstein J Org Chem. 2017 May 30;13:1039–49. doi: 10.3762/bjoc.13.103 (PMC5480333; doi:10.3762/bjoc.13.103)
Supplement: File 1 — HRESIMS, 1D and 2D NMR spectra of compounds 1–3; energies, populations, and key torsion angles of low-energy conformers of compounds 1–3 by theoretical computations. [file Beilstein_J_Org_Chem-13-1039-s001.pdf]

Supporting Information

for

Cycloheximide congeners produced by *Streptomyces* sp. SC0581 and photoinduced interconversion between (*E*)- and (*Z*)-2,3-dehydroanhydrocycloheximides

Li Yang[1,2], Ping Wu[1], Jinghua Xue[1], Huitong Tan[1], Zheng Zhang[1] and Xiaoyi Wei*[1]

Address: [1]Key Laboratory of Plant Resources Conservation and Sustainable Utilization/ Guangdong Provincial Key Laboratory of Digital Botanical Garden, South China Botanical Garden, Chinese Academy of Sciences, Xingke Road 723, Tianhe District, Guangzhou 510650, China and [2]University of Chinese Academy of Sciences, Yuquanlu 19A, Beijing 100049, China.

Email: Xiaoyi Wei[*]-wxy@scbg.ac.cn.

[*]Corresponding author

HRESIMS, 1D and 2D NMR spectra of compounds 1–3; energies, populations, and key torsion angles of low-energy conformers of compounds 1–3 by theoretical computations

Table of contents

1. Theoretical computations S3

Table S1. Energies, populations, and key torsion angles of low-energy conformers of **1** S3

Figure S1. Comparison of the experimental ECD spectrum of **1** with the calculated spectra of (4*R*,6*R*,α*S*)-**1** and (4*S*,6*S*,α*R*)-**1** S4

Figure S2. Comparison of the experimental ECD spectrum of **1** with the calculated spectra of (4*R*,6*R*,α*R*)-**1** and (4*S*,6*S*,α*S*)-**1** S4

Table S2. Energies, populations, and key torsion angles of low-energy conformers of **2** and **3** S5

2. NMR spectra and HRESIMS S6

Figure S3. ^{1}H NMR (500 MHz) spectrum of compound **1** in CD_3OD S6

Figure S4. ^{13}C NMR (125 MHz) spectrum of compound **1** in CD_3OD S6

Figure S5. HSQC spectrum of compound **1** in CD_3OD S7

Figure S6. ^{1}H,^{1}H COSY spectrum of compound **1** in CD_3OD S7

Figure S7. HMBC spectrum of compound **1** in CD_3OD S8

Figure S8. NOESY spectrum of compound **1** in CD_3OD S8

Figure S9. HRESIMS of compound **1** S9

Figure S10. ^{1}H NMR (500 MHz) spectrum of compounds **2**/**3** in CD_3OD S10

Figure S11. ^{13}C NMR (125 MHz) spectrum of compounds **2**/**3** in CD_3OD S10

Figure S12. HSQC spectrum of compounds **2**/**3** in CD_3OD S11

Figure S13. ^{1}H,^{1}H COSY spectrum of compounds **2**/**3** in CD_3OD S11

Figure S14. HMBC spectrum of compounds **2**/**3** in CD_3OD S12

Figure S15. NOESY spectrum of compounds **2**/**3** in CD_3OD S12

Figure S16. HRESIMS of compounds **2**/**3** S13

1. Theoretical computations

Table S1. Relative thermal energies (Δ*E*, kcal/mol)[a], and relative free energies (Δ*G*, kcal/mol)[a], equilibrium populations (P, %)[b], and key torsion angles (ϕ, degree) of low-energy conformers of **1** in MeOH

conformer	$\phi_{C3\text{-}C4\text{-}C5\text{-}C6}$	$\phi_{H6\text{-}C6\text{-}C\alpha\text{-}H\alpha}$	Δ*E*	Δ*G*	P
(4*R*,6*R*,α*S*)-**1**					
S-**1a1**	-49.45	-60.90	0.00	0.02	25.8
S-**1a2**	-49.48	-61.12	0.65	0.58	10.1
S-**1a3**	-49.75	-62.98	0.76	0.78	7.2
S-**1a4**	-49.77	-64.17	1.34	1.15	3.9
S-**1a5**[c]	-49.79	-61.51	1.50	2.11	0.8
S-**1b1**	-46.27	55.65	0.56	0.00	26.9
S-**1b2**	-46.25	54.89	0.89	0.68	8.6
S-**1b3**	-46.31	55.31	1.16	0.99	5.1
S-**1b4**	-46.50	54.46	0.79	1.03	4.7
S-**1b5**[c]	-46.26	56.72	1.94	1.67	1.6
S-**1b6**[c]	-46.26	55.23	1.45	1.78	1.3
S-**1c1**[c]	-48.10	159.61	2.28	1.61	1.8
S-**1c2**[c]	-47.98	163.21	2.42	1.92	1.1
S-**1c3**[c]	-47.60	164.54	3.08	2.08	0.8
(4*R*,6*R*,α*R*)-**1**					
R-**1a1**	-48.24	174.44	0.00	0.00	58.1
R-**1a2**	-48.11	174.07	1.06	1.02	10.3
R-**1a3**	-48.46	173.83	1.06	1.20	7.7
R-**1a4**	-48.13	174.97	1.35	1.84	2.6
R-**1a5**	-48.29	173.22	2.06	1.97	2.1
R-**1a6**[c]	-48.55	173.01	1.99	2.37	1.1
R-**1b1**	-46.12	-81.43	1.42	1.26	6.9
R-**1b2**[c]	-46.04	-79.73	2.17	2.10	1.7
R-**1b3**[c]	-46.10	-80.46	2.56	2.39	1.0
R-**1c1**	-48.81	63.24	2.27	1.42	5.3
R-**1c2**	-48.89	63.88	2.55	1.89	2.4

[a] At the B3LYP-D3/def2-TZVP level, in kcal/mol. [b] From Δ*G* values at 298.15 K.

[c]Conformer not used for ECD/ TDDFT calculations.

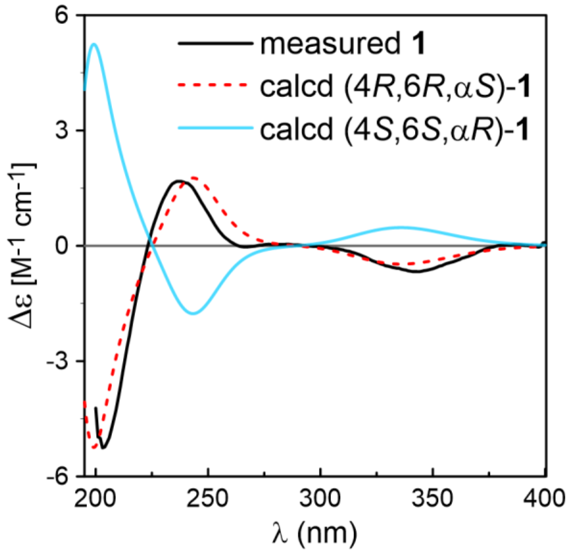

Figure S1. Comparison of the experimental ECD spectrum of **1** with the M11/TZVP calculated spectra of (4*R*,6*R*,α*S*)-**1** and (4*S*,6*S*,α*R*)-**1** in MeOH (σ = 0.38 eV, shift = +15, scaling factor = 0.50).

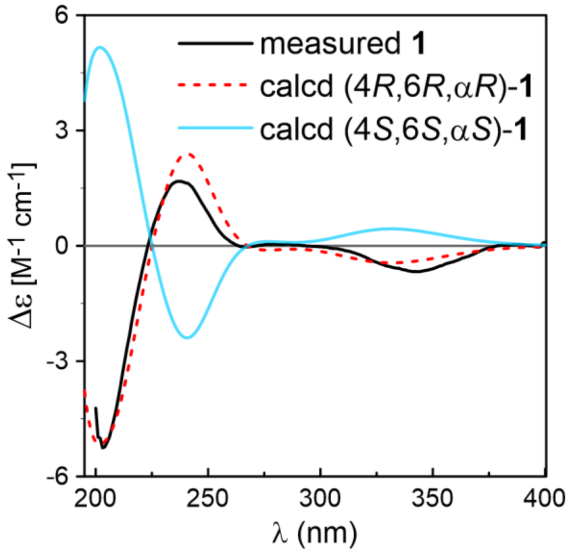

Figure S2. Comparison of the experimental ECD spectrum of **1** with the M11/TZVP calculated spectra of (4*R*,6*R*,α*R*)-**1** and (4*S*,6*S*,α*S*)-**1** in MeOH (σ = 0.38 eV, shift = +10 nm, scaling factor = 1.0).

Table S2. Relative thermal energies (Δ*E*, kcal/mol)[a], and relative free energies (Δ*G*, kcal/mol)[a], equilibrium populations (P, %)[b], and key dihedral angles (ϕ, degree) of low-energy conformers of **2** and **3** in MeOH

conformer	$\phi_{C3\text{-}C4\text{-}C5\text{-}C6}$	$\phi_{C6\text{-}C\alpha\text{-}C\beta\text{-}C4'}$	Δ*E*	Δ*G*	P
(4*R*)-**2**					
**2a**	-48.32	105.45	0.00	0.00	15.8
**2b**	-47.27	-104.97	0.26	0.11	13.1
**2c**	-48.11	85.29	0.80	0.27	10.0
**2d**	48.39	105.10	0.55	0.28	9.9
**2e**	48.55	-118.48	0.15	0.54	6.3
**2f**	48.05	-108.81	0.95	0.59	5.9
**2g**	-46.85	-84.15	0.87	0.64	5.3
**2h**	-48.01	99.43	0.55	0.72	4.7
**2i**	-46.91	-97.81	0.80	0.80	4.1
**2j**	48.15	81.99	1.18	0.85	3.7
**2k**	49.08	-83.30	0.83	1.16	2.2
**2l**[c]	48.27	98.62	1.11	1.32	1.7
(4*R*)-**3**					
**3a**	48.77	121.83	0.44	0.00	39.5
**3b**	-47.64	106.80	0.00	0.79	10.4
**3c**	-47.75	-95.66	0.53	0.89	8.7
**3d**	-47.63	86.64	0.38	1.02	7.0
**3e**	48.74	-106.18	0.50	1.33	4.2
**3f**	48.63	-85.95	0.85	1.47	3.3
**3g**	-48.50	-72.80	1.26	1.64	2.5
**3h**	-47.58	-91.79	1.13	1.66	2.4
**3i**	-47.66	104.16	0.81	1.67	2.3
**3j**[c]	50.03	72.24	1.39	2.01	1.3
**3k**[c]	48.71	-103.76	1.24	2.27	0.9
**3l**[c]	48.15	92.15	1.26	2.29	0.8

[a] At the B3LYP-D3/def2-TZVP level, in kcal/mol. [b]From Δ*G* values at 298.15 K. [c]Conformer not used for ECD/ TDDFT calculations.

2. NMR spectra and HRESIMS

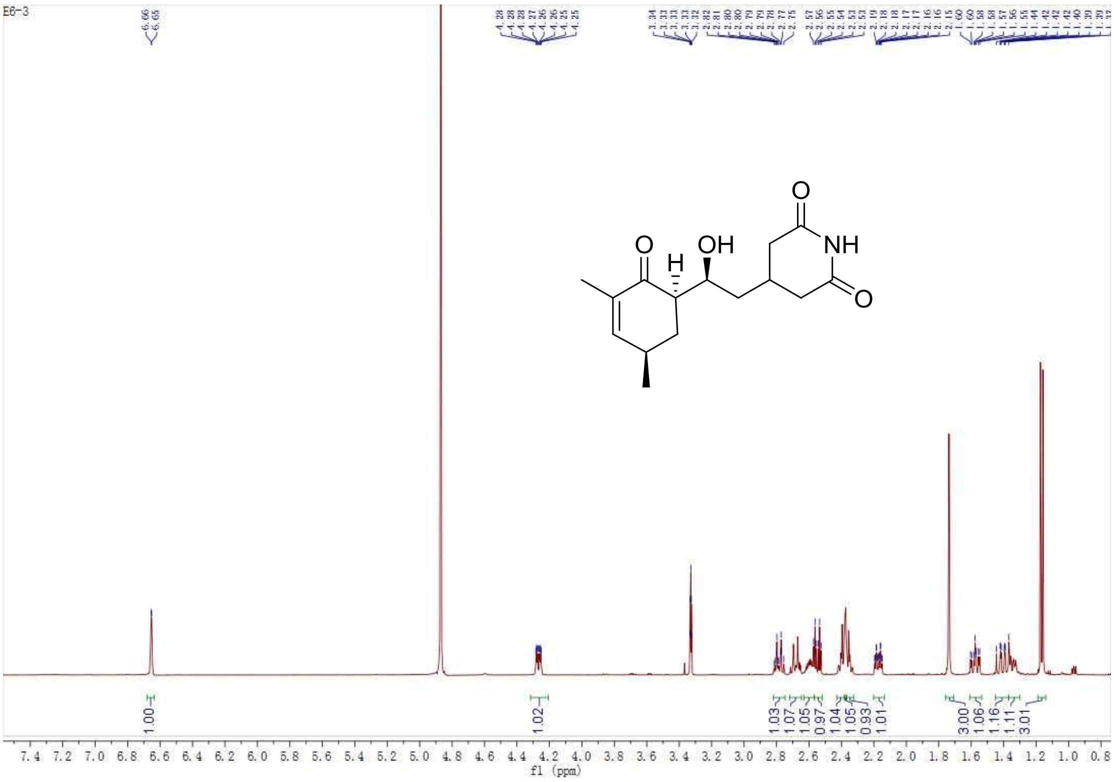

Figure S3. ^{1}H NMR (500 Hz) spectrum of compound **1** in CD_3OD.

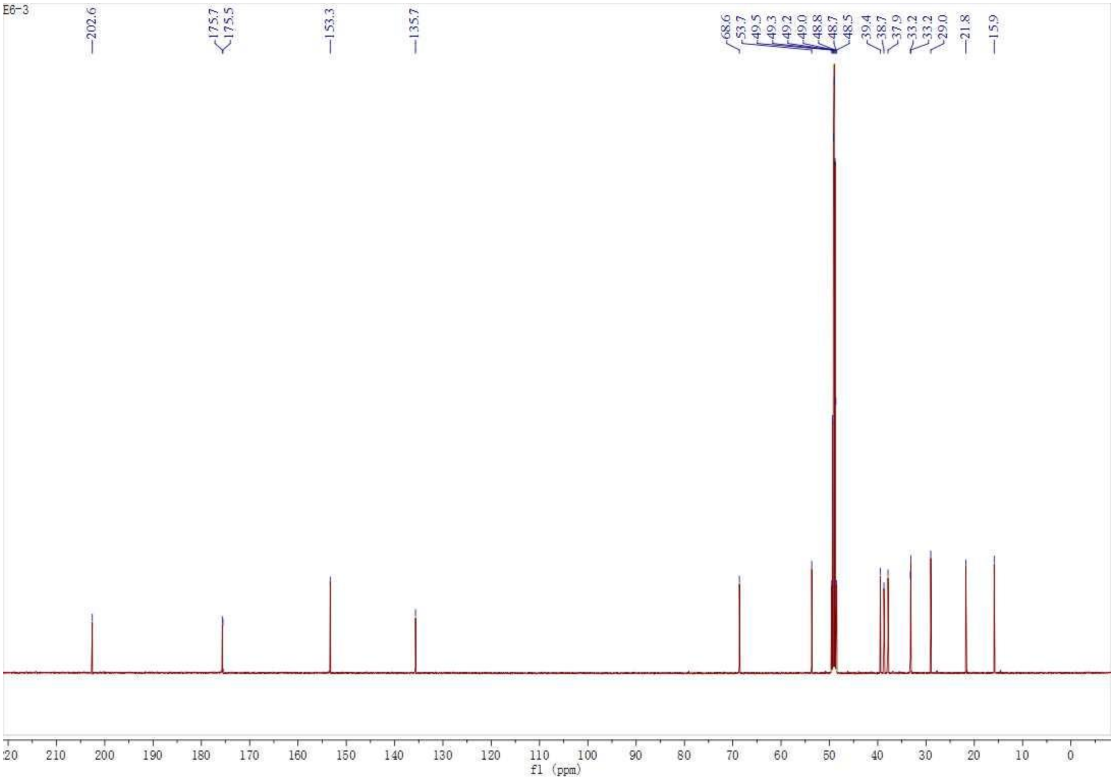

Figure S4. ^{13}C NMR (125 Hz) spectrum of compound **1** in CD_3OD.

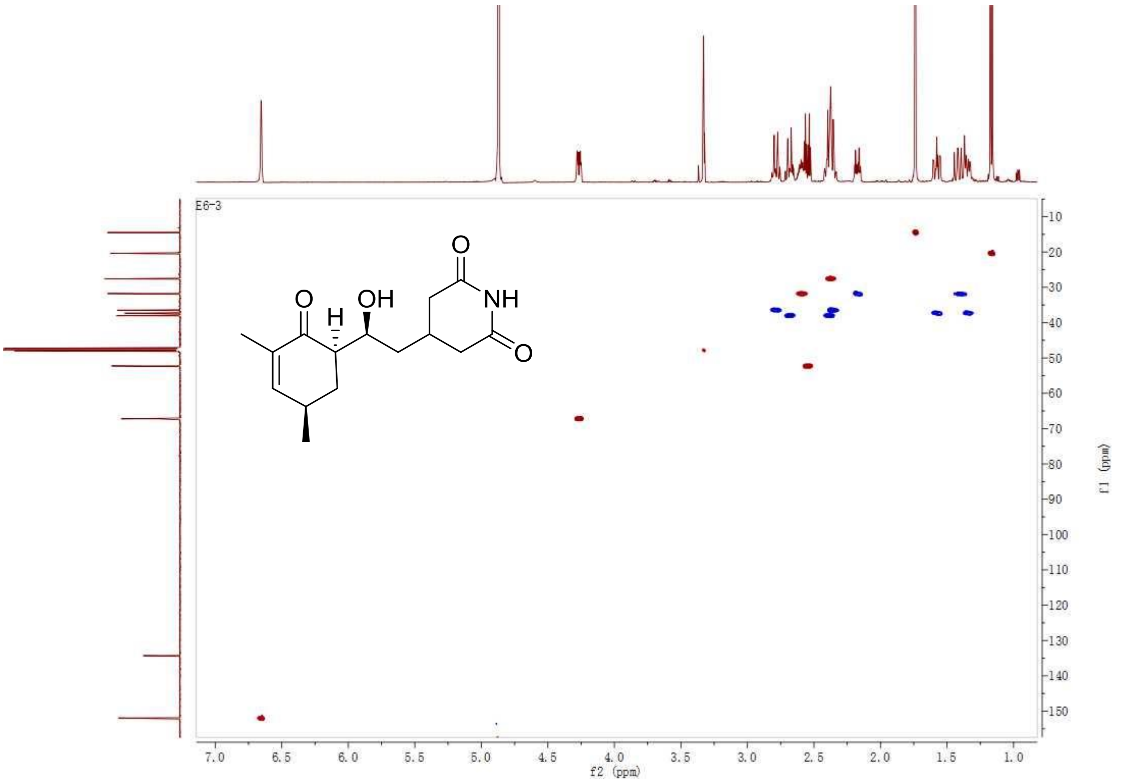

Figure S5. HSQC spectrum of compound **1** in CD_3OD.

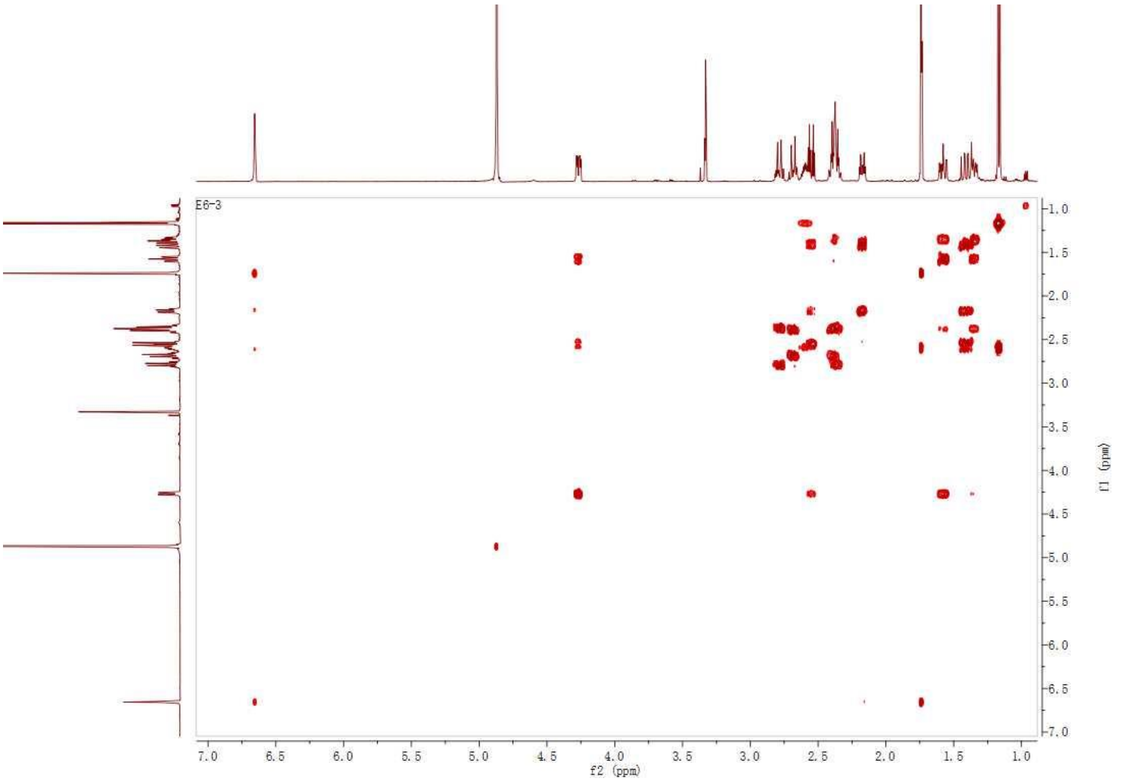

Figure S6. ^1H-^1H COSY spectrum of compound **1** in CD_3OD.

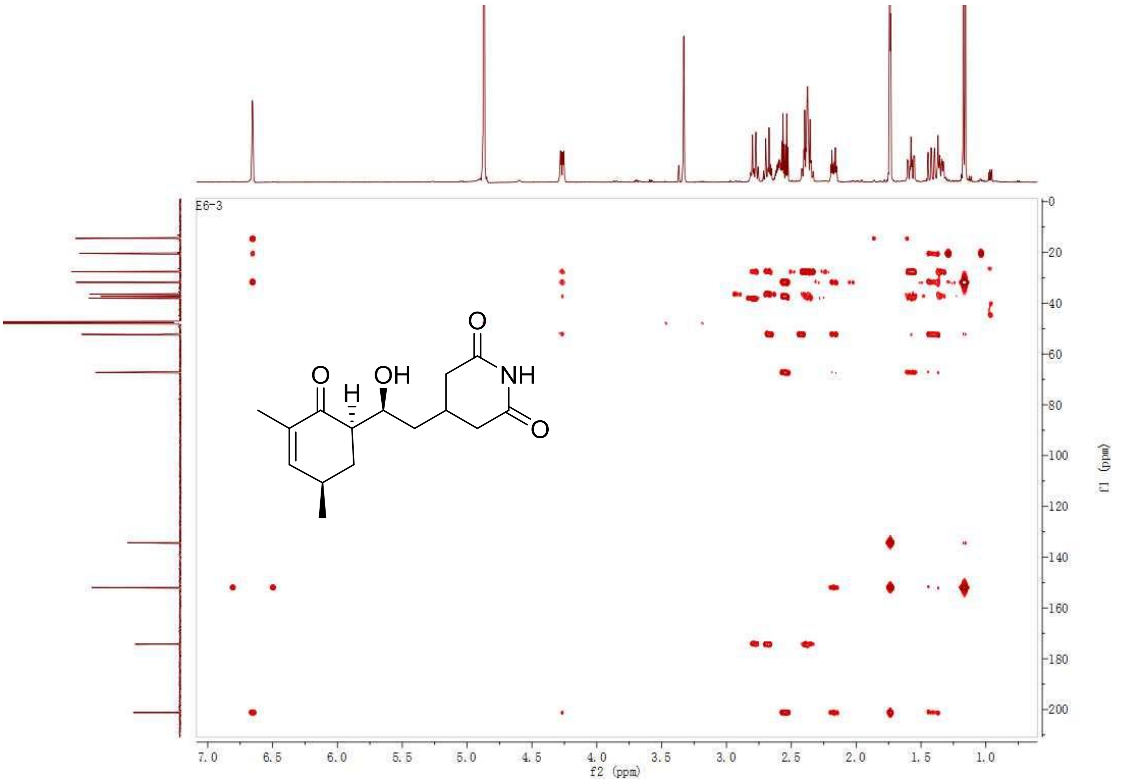

Figure S7. HMBC spectrum of compound **1** in CD_3OD.

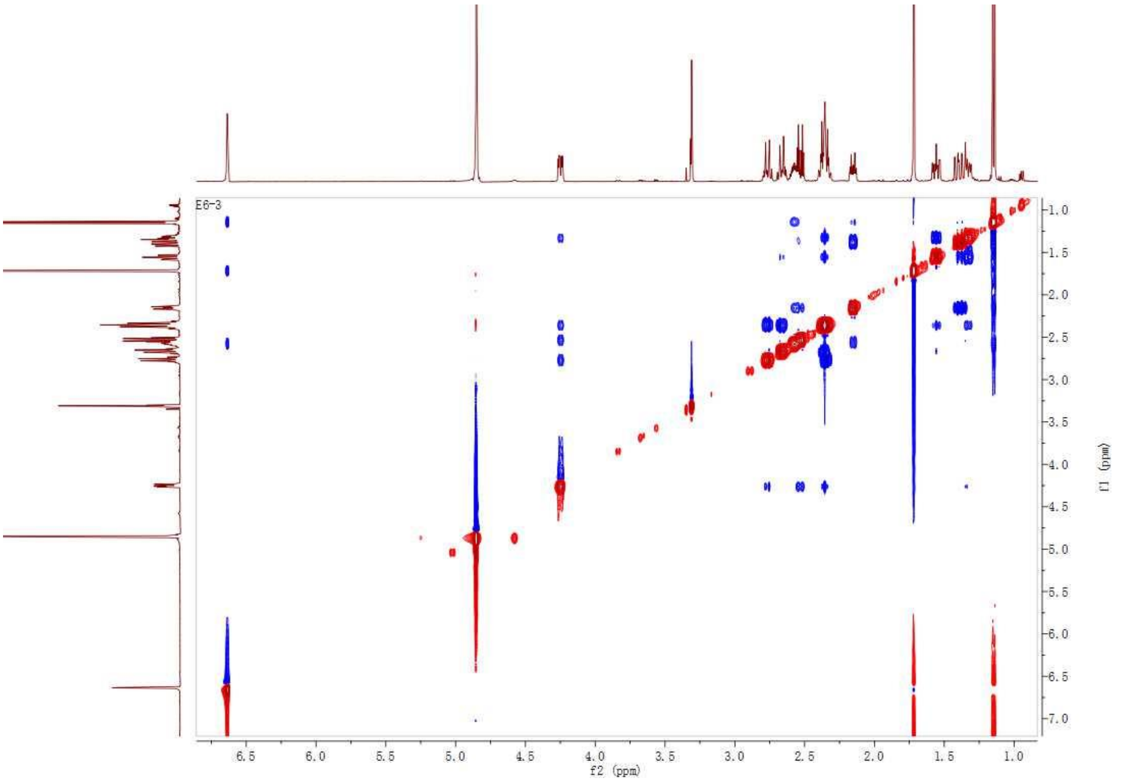

Figure S8. NOESY spectrum of compound **1** in CD_3OD.

Mass Spectrum SmartFormula Report

Analysis Info			
Analysis Name	D:\Data\MS\data\201408\wuping_E6-3_pos.d	Acquisition Date	8/28/2014 10:36:47 AM
Method	POS_100-2000_Dirrect Infusion.m	Operator	SCSIO
Sample Name	SCSIO	Instrument / Ser#	maXis 29
Comment			

Acquisition Parameter

Source Type	ESI	Ion Polarity	Positive	Set Nebulizer	0.3 Bar
Focus	Not active	Set Capillary	4500 V	Set Dry Heater	180 °C
Scan Begin	100 m/z	Set End Plate Offset	-500 V	Set Dry Gas	4.0 l/min
Scan End	2000 m/z	Set Collision Cell RF	2000.0 Vpp	Set Divert Valve	Waste

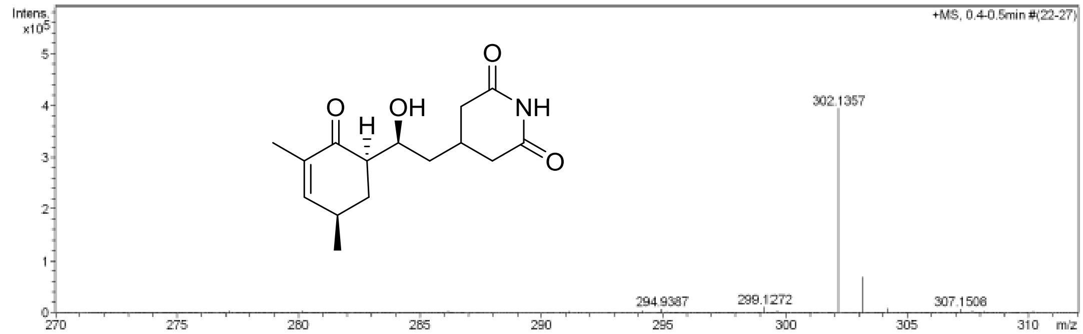

Meas. m/z	#	Formula	m/z	err [ppm]	Mean err [ppm]	rdb	N-Rule	e⁻ Conf	mSigma	Std I	Std Mean m/z	Std I VarNorm	Std m/z Diff	Std Comb Dev
280.1536	1	C 15 H 22 N O 4	280.1543	2.5	3.5	5.5	ok	even	23.9	42.2	1.5	17.0	0.8	842.7
302.1357	1	C 15 H 21 N Na O 4	302.1363	1.9	2.0	5.5	ok	even	5.4	9.0	0.6	3.8	0.1	842.7
334.1627	1	C 16 H 25 N Na O 5	334.1625	-0.5	0.5	4.5	ok	even	241.0	313.1	0.5	128.7	0.7	855.0
645.3344	1	C 32 H 50 N 2 Na O 10	645.3358	2.1	1.6	8.5	ok	even	25.8	44.5	2.0	24.1	0.5	828.7

Figure S9. HRESIMS of compound **1.**

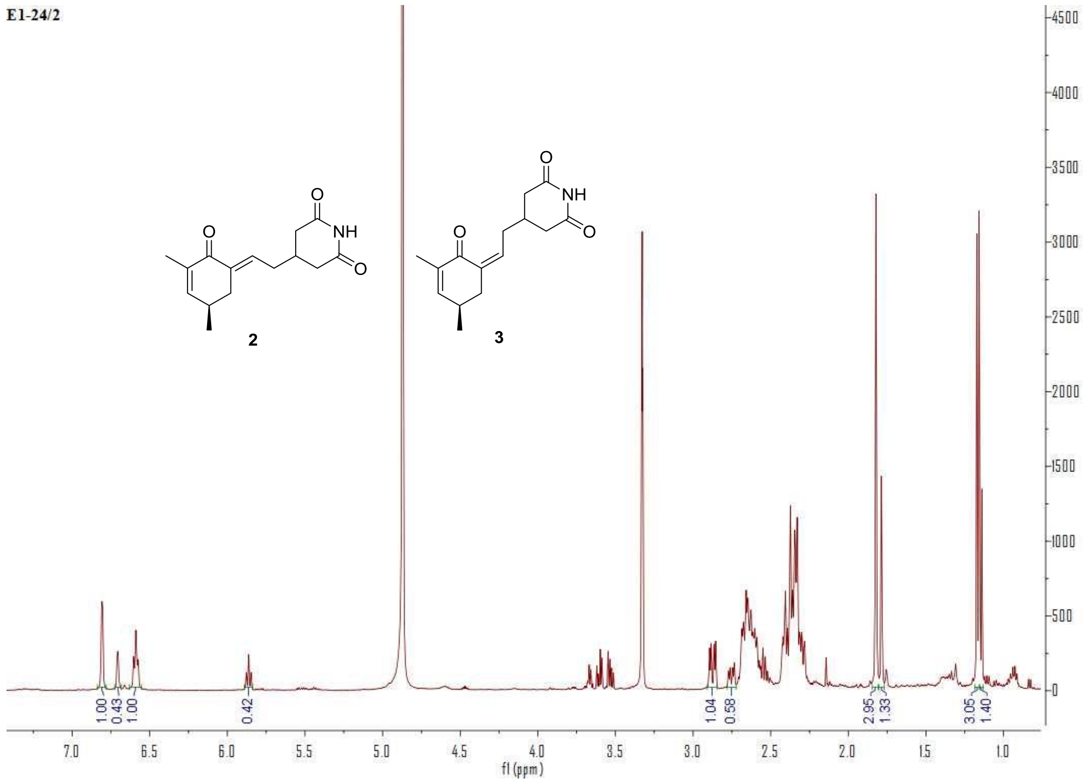

Figure S10. ^{1}H NMR (500 MHz) spectrum of compounds **2**/**3** in CD_3OD.

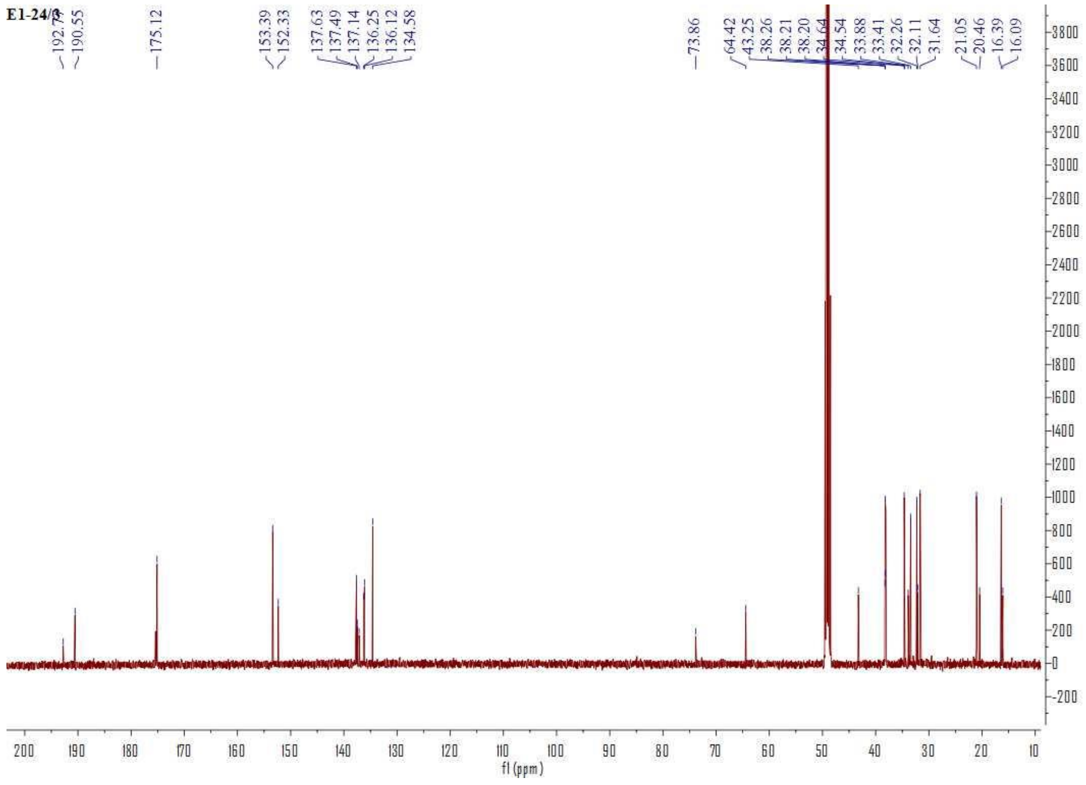

Figure S11. ^{13}C NMR (125 MHz) spectrum of compounds **2**/**3** in CD_3OD.

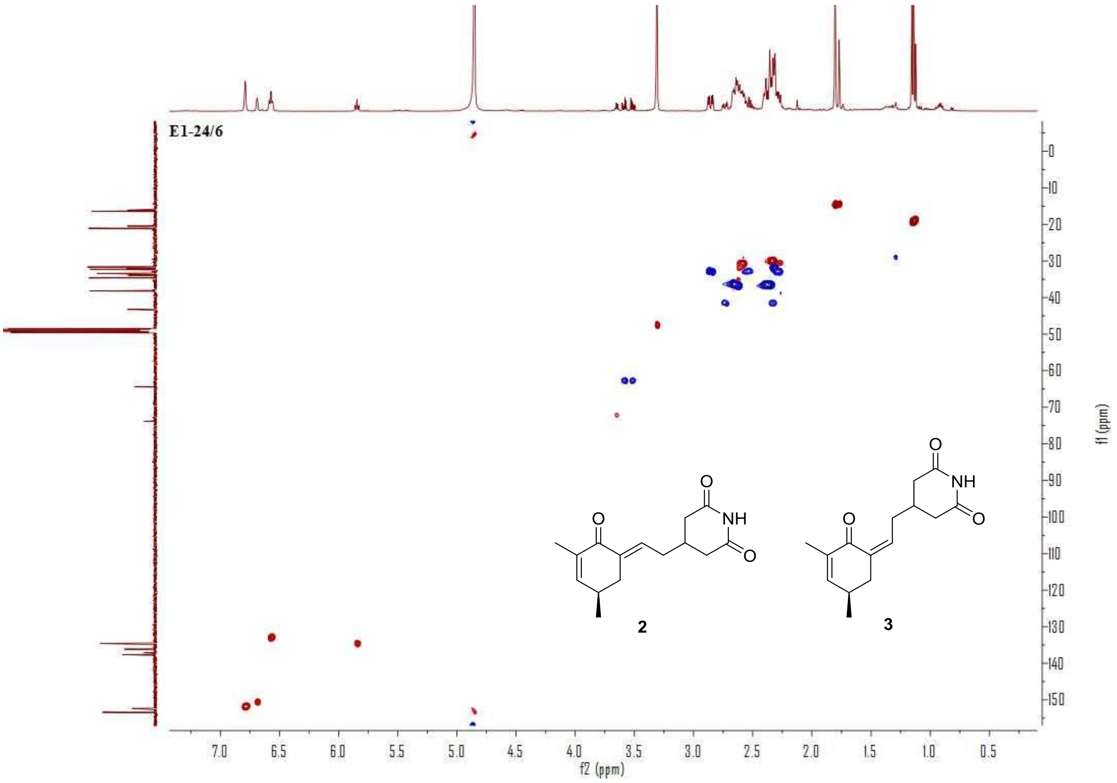

Figure S12. HSQC spectrum of compounds **2**/**3** in CD_3OD.

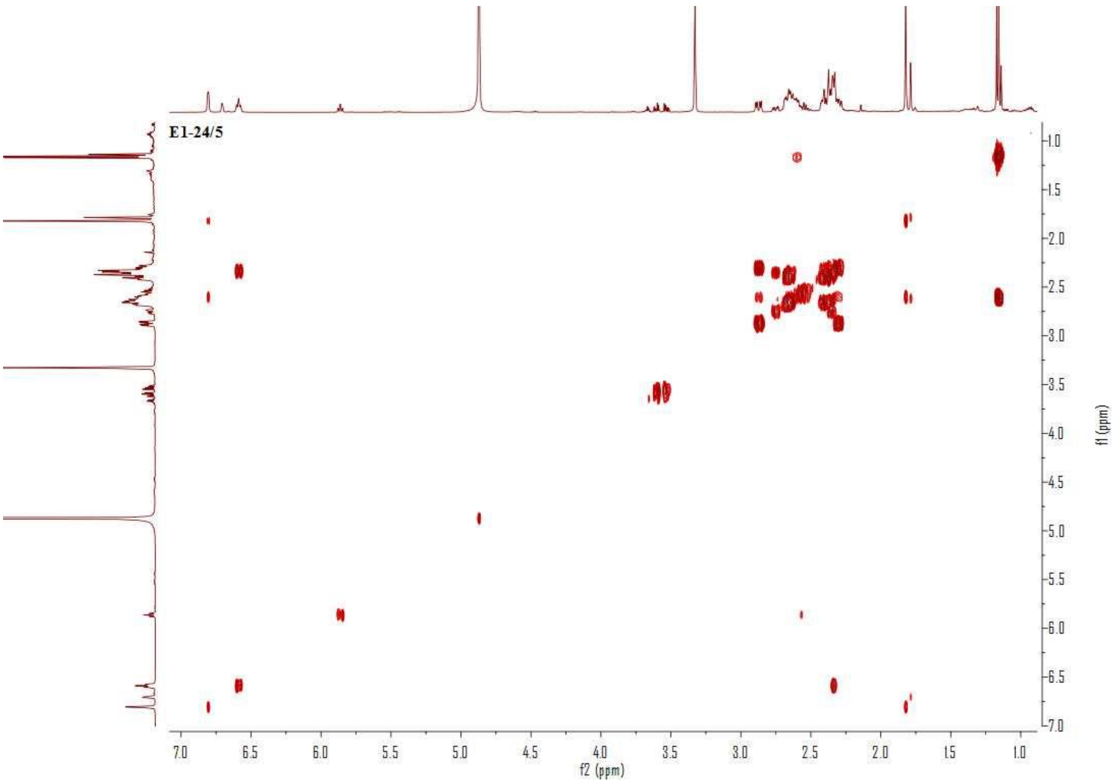

Figure S13. $^1H,^1H$ COSY spectrum of compounds **2**/**3** in CD_3OD.

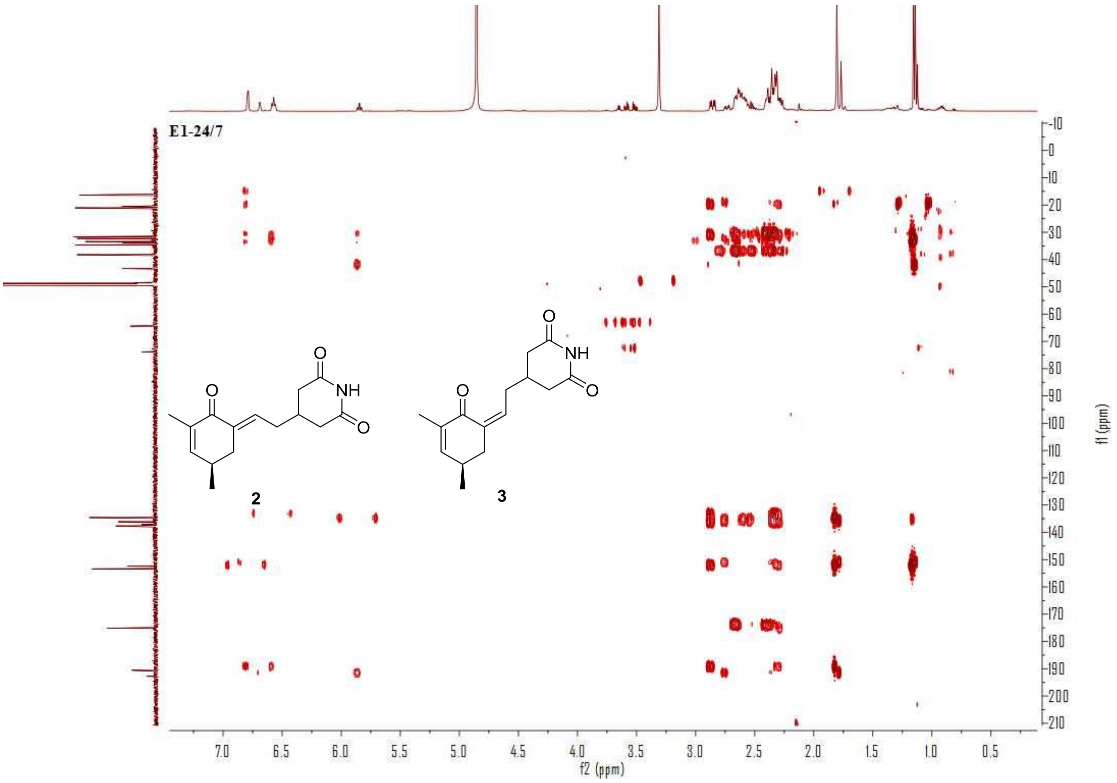

Figure S14. HMBC spectrum of compounds **2**/**3** in CD_3OD.

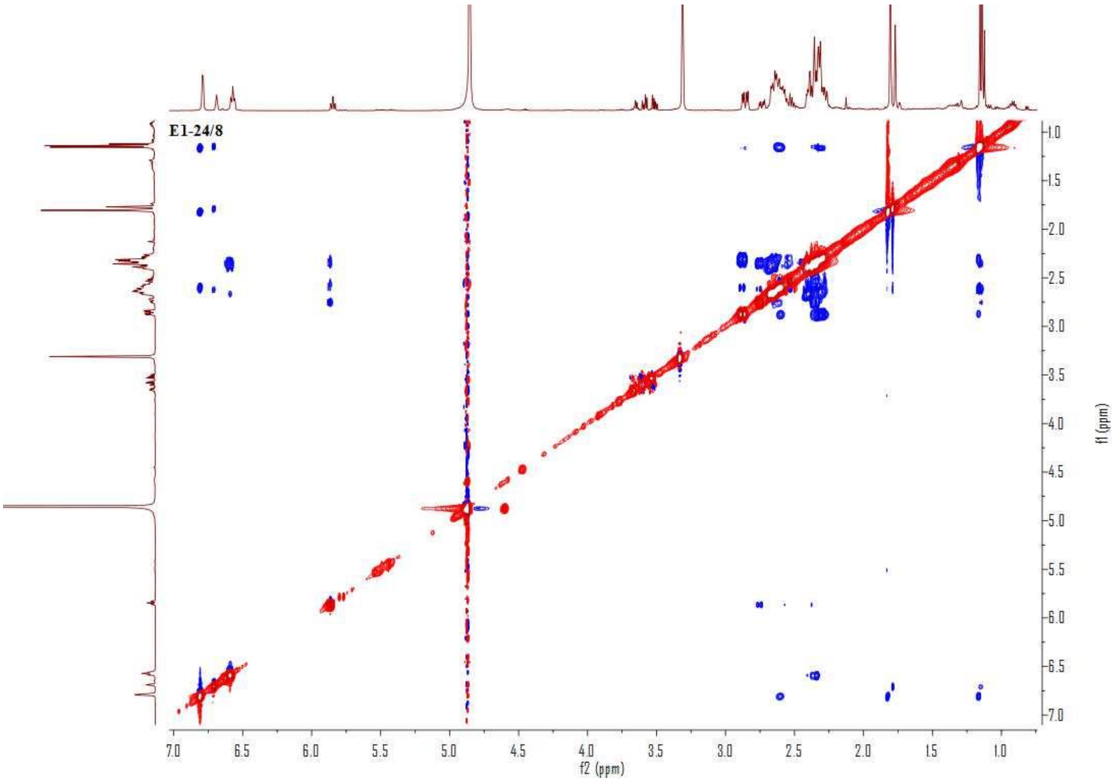

Figure S15. NOESY spectrum of compounds **2**/**3** in CD_3OD.

Mass Spectrum SmartFormula Report

Analysis Info

Analysis Name	D:\Data\MS\data\201506\E1-24_2_01_64.d	Acquisition Date	6/17/2015 10:04:34 PM
Method	lc_direct infusion_pos_100-1000mz.m	Operator	SCSIO
Sample Name	E1-24	Instrument / Ser#	maXis 29
Comment			

Acquisition Parameter

Source Type	ESI	Ion Polarity	Positive	Set Nebulizer	0.4 Bar
Focus	Active	Set Capillary	4500 V	Set Dry Heater	180 °C
Scan Begin	100 m/z	Set End Plate Offset	-500 V	Set Dry Gas	4.0 l/min
Scan End	2000 m/z	Set Collision Cell RF	800.0 Vpp	Set Divert Valve	Waste

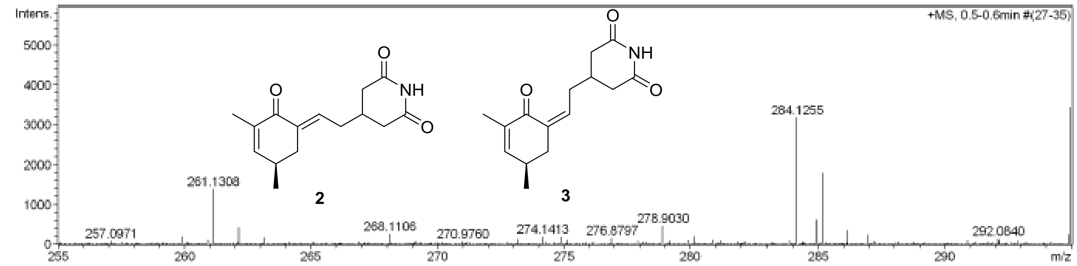

Meas. m/z	#	Formula	Score	m/z	err [ppm]	err [mDa]	mSigma	rdb	e⁻ Conf	N-Rule
262.1455	1	C 15 H 20 N O 3	100.00	262.1438	-6.5	-1.7	153.9	6.5	even	ok
284.1255	1	C 15 H 19 N Na O 3	100.00	284.1257	0.6	0.2	234.8	6.5	even	ok

Figure S16. HRESIMS of compounds **2**/**3**.
